# Commissioning a 50–100 kV X‐ray unit for skin cancer treatment

**DOI:** 10.1120/jacmp.v16i2.5182

**Published:** 2015-03-08

**Authors:** Ren‐Dih Sheu, Allison Powers, Yeh‐Chi Lo

**Affiliations:** ^1^ Department of Radiation Oncology Mount Sinai School of Medicine New York NY

**Keywords:** low‐energy X‐rays, dosimetry, calibration, ionization chambers, GafChromic films

## Abstract

This study provides the authors' experience along with dosimetric data from the commissioning of two Sensus SRT‐100 50‐100 kV X‐ray units. Data collected during the commissioning process included: a) HVL, b) output (dose rate), c) applicator cone factors, and d) percentage depth dose. A Farmer‐type chamber (PTW‐N23333), and a thin‐window parallel plate ion chamber (PTW‐N23342) were used for dose rate measurements and dose profiles were measured with EBT3 GafChromic film. The average HVL values for 50, 70, and 100 kV of the two treatment units were found to be 0.52, 1.15, and 2.20 mm Al, respectively. The HVL's were 5%–9% lower when measured with the Farmer chamber, as compared to measurements with the parallel plate chamber, for energies of 70 and 100 kV. Dose rates were also measured to be 3%–4% lower with the Farmer chamber. The dose rate variation between the two units was found to be 2%–9% for 50, 70, and 100 kV. The dose uniformity over a circle of 2 cm diameter was within 4% in four cardinal directions; however, the dose profiles for the 5 cm applicator were nonuniform, especially in the cathode–anode direction. Measurements indicated as much as 15% lower dose for the 50 kV beam at field edge on the anode side, when normalized to the center. The crossline profile was relatively more symmetric, with a maximum deviation of 10% at the field edge. All ion chamber readings agreed with film measurements within 3%. The nonuniform profile produced by these units may introduce uncertainty in dose rate measurements, especially for larger applicators. Since there is no intrinsic tool (crosshair or field light) for alignment with the beam axis, the user should take care when positioning the chamber for output measurements. The data obtained with a Farmer‐type chamber should be used cautiously and as a reference only for the SRT‐100 X‐ray treatment unit.

PACS number: 87.53.Bn

## I. INTRODUCTION

Superficial X‐rays have been used since the 1950s to treat basal and squamous cell carcinomas.^(1)^ Electron beam treatments produce comparable results to soft X‐rays in the treatment of these diseases; however, the implementation of soft X‐rays is much simpler and cost‐effective. Dermatology practices have become aware of this and consequently, there is widespread use of these systems by dermatologists.^(2)^ For treating most skin lesions, particularly those around the eyes, superficial X‐rays are preferable due to ease in field shaping, as well as superior protection from scatter radiation to sensitive nearby structures. Considering the ease of use, quality of treatment, and cost effectiveness, the acquisition of a superficial radiation unit in a modern radiation oncology department expands the lineup of treatment modalities and, for many cases, gives more suitable treatments.

Since superficial radiation has been used for more than 50 years, many existing systems in radiation oncology departments have been in use for quite some time and are antiquated by today's standards. The SRT‐100 is a recently developed mobile superficial therapy system by Sensus Healthcare, which can be controlled remotely, delivers dose rates comparable to electrons, and provides several safety features to meet modern requirements.^(3)^

To commission the SRT‐100 skin treatment machine, we followed the protocol for 40‐300 kV X‐ray beam dosimetry, AAPM Task Group 61.^(4)^ TG‐61 describes the details of reference dosimetry for low‐energy X‐rays. A study by Rong and Welsh^(5)^ can also serve as a practical source of useful information and detailed procedures for the calibration and commissioning of low energy X‐ray skin treatment machines. Devising an appropriate commissioning procedure for a superficial treatment unit is important as the accuracy of measurements are dependent on several factors, such as chamber calibration factor, chamber position, and target to surface distance. Uncertainties in dose rate measurement can be large at low kilovoltage energies. In particular, the accuracy of ion chamber positioning will affect dose calibration due to nonuniformities across the field. The lack of a tool for alignment or reproducible positioning of the chamber for output measurements compounds this difficulty.

This work aimed to provide procedures and dosimetric data from the commissioning of two SRT‐100 units. We also investigated the uncertainty of dosimetric data for this skin machine.

## II. MATERIALS AND METHODS

Two superficial treatment units manufactured by Sensus Healthcare (Boca Raton, FL) were studied in this work. Both are the same model, SRT‐100, and have three nominal X‐ray energies, 50, 70, and 100 k V, as well as six applicators ranging from 1.5 to 5 cm diameter for clinical use. Each applicator consists of two parts: a metal cone and a replaceable translucent shield tip. The treatment applicator is designed such that the translucent tip should be placed flush against the skin or treatment site for a sharp penumbra. The clear shielding at the tip of the applicator provides visual verification of the treatment area and a fixed TSD. The target‐to‐skin distance is 15 cm for all the applicators in this study. The filters are built‐in and automatically move into place upon energy selection. The unit uses a standard wall socket plug with 120‐V AC power. The compact size (76.2×76.2 cm footprint) allows us to store the unit inside a vault with a linear accelerator.

### A. Area survey

The unit was installed in an existing 6 MV linac treatment room; hence, no extra construction was needed. All the patient monitoring/safety systems are shared with MV treatments from the linear accelerator, so no additional safety device was required. A radiation survey was performed before the commissioning process and readings were at background level outside the treatment room.

### B. Dosimetry equipment

For beam dosimetry, we followed the protocol published by AAPM Task Group 61. The energy range for this specific unit is considered “low energy” (or superficial) and the in‐air method was applied. The dose to water at the surface of a water phantom was obtained based on an in‐air measurement using an ion chamber also calibrated in air. No phantom was necessary for output measurements.

A Farmer‐type chamber (PTW‐N23333; Freiburg, Germany) and a thin‐window parallel plate (PP) ion chamber (PTW‐N23342) with IBA Dose1 (IBA Dosimetry, Schwarzenbruck, Germany) and PTW UNIDOS electrometers were used in this study.

Data collected during commissioning were: a) HVL, b) dose rate (absolute output) at 15 cm target‐to‐skin distance (TSD) in air under 5 cm applicator, c) depth dose, d) dose profile, e) dose outside the applicator, and f) applicator cone factors. Measured data were compared with the values provided by the vendor.

**Figure 1:**
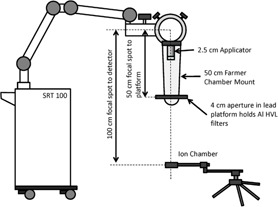
Half value layer measurement setup.

The 50 cm Farmer chamber mount that came with the treatment unit was used for HVL measurement with the setup recommended in TG‐61. As shown in Fig. 1, the distance from the target spot to the diaphragm and detector were 50 cm and 100 cm, respectively.

The air kerma calibration coefficients ($N_{k}$) for the 50 k V, 70 kV, and 100 kV were deduced by performing linear interpolation or extrapolation from the UW‐ADCL calibration report listed in 1, 2 for the Farmer and parallel plate chambers, respectively, to the user‐determined values of HVL.

Temperature and pressure were measured by using a Traceable Lollipop digital thermometer Thomas Scientific, Swedesboro, NJ) and a Druck DPI 705 digital hand held pressure indicator (GE Measurement & Control, Boston, MA). The temperature and pressure correction (CTP) was applied for the dose rate calibration.

Dose rate was measured at 15 cm TSD, using the 5 cm diameter applicator. The setups for the Farmer and parallel plate ion chamber are shown in 2, 3, respectively. Since there is no built‐in alignment tool or standardized holder to secure the detector position, the user should be aware of the uncertainty due to chamber position and exhibit caution when positioning the chamber. A 1 mm deviation of TSD results in a 1.3% output uncertainty by the inverse square law, due to the short TSD (15 cm).

**Table 1:** PTW‐N23333 Chamber ADCL calibration coefficient

*Beam*	*kVp*	*HVL (mm)*	$N_{k}$ *(cGy/nC)*	*Uncertainty*
W/Al	70	2.31 Al	4.834	1.5%
W/Al	50	1.03 Al	5.017	1.5%
W/Al	30	0.35 Al	5.511	1.5%

**Table 2:** PTW‐N23342 PP chamber ADCL calibration coefficient

*Beam*	*kVp*	*HVL (mm)*	$N_{k}$ *(cGy/nC)*	*Uncertainty*
UW80‐L	80	1.83 Al	101.2	1%
UW40‐L	40	0.503 Al	103.7	1%

**Figure 2:**
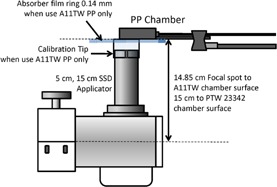
Absolute measurement setup for PP chamber and 5 cm dia./15 cm SSD application.

**Figure 3:**
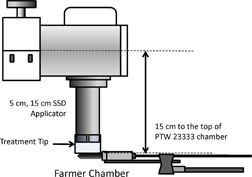
Absolute measurement setup for Farmer‐type thimble chamber and 5 cm dia./15 cm SSD application.

**Figure 4:**
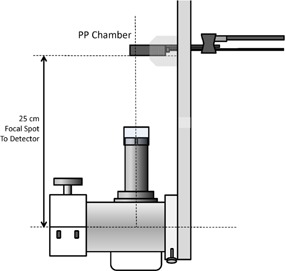
Relative applicator cone factor measurement setup.

The applicator cone factors were normalized to the dose from 5 cm applicator at 25 cm TSD (Fig. 4). The parallel plate chamber was used for this measurement because the size of this chamber is small enough (0.02 cm^3^) to measure the smallest applicator (1.5 cm) at 25 cm TSD.

The backscatter factors ($B_{w}$) were obtained from TG‐61 protocol Table V by using the HVL measurement obtained during commissioning. The $B_{w}$ data points were based on the study by Grosswendt.^(6)^

The ratio of mass energy‐absorption coefficients water to air, free in air, to convert air kerma to water kerma ${\left[{\left(\frac{\bar{\mu_{en}}}{\rho }\right)}_{air}^{w}\right]}_{air}$ were obtained from TG‐61 protocol (B1.1 Table IV) or each kV operating voltage.

Based on TG‐61, the absorbed dose to water at the surface for low‐energy X‐rays can be determined according to

(1) $$D_{w,z=0}=MN_{k}B_{w}{\left[{\left(\frac{\bar{\mu_{en}}}{\rho }\right)}_{air}^{w}\right]}_{air}$$

**Figure 5:**
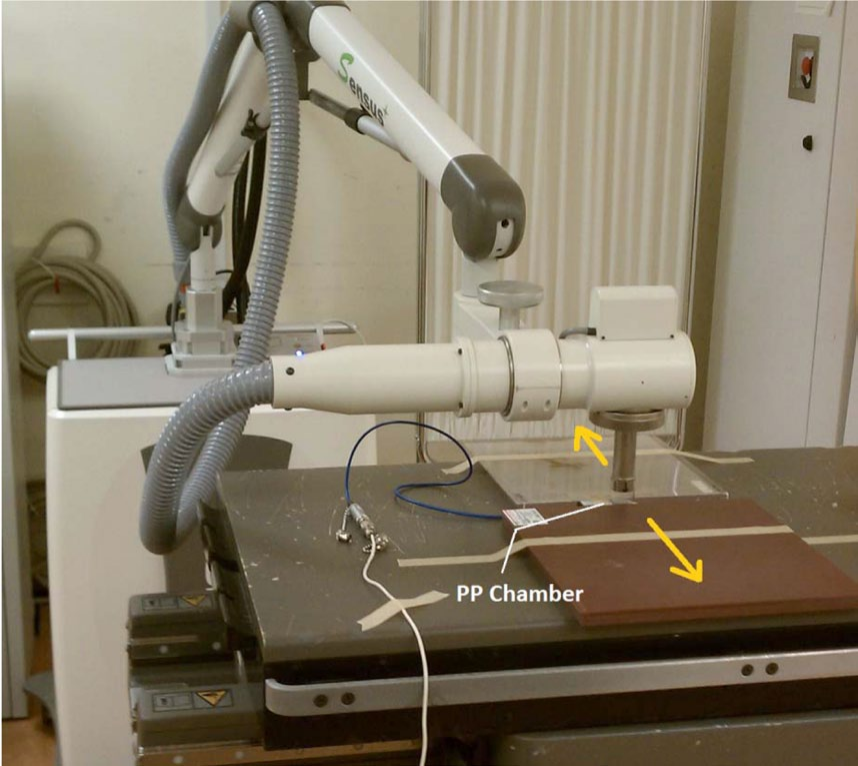
Setup for ion chamber positioning measurement (dose profile and outside dose) by using Varian Exact couch.

To investigate the impact of chamber position on dose rate measurement, we used both GafChromic film (EBT3) (International Specialty Products, Wayne, NJ) and the parallel plate ion chamber to measure the dose profiles. The dose response of EBT3 film was calibrated using a 3‐channel technique.^(7)^ A maximum applicator size of 5 cm was used in this study. The readings were limited to measurements at 1 and 2 cm away from the central axis in four directions (± parallel and perpendicular to cathode–anode axis) due to the size of the ion chamber. The ion chamber was positioned by using Varian Exact couch (Varian Medical Systems, Palo Alto, CA) within 1 mm accuracy of movement, as shown in Fig. 5.

The parallel plate ion chamber was used to measure the surface dose (relative to center) outside the applicator at the distances of 1 cm and 2 cm from the field edge. The chamber was placed in the Solid Water phantom and positioned using the Varian Exact couch.

The percentage depth‐dose curves were generated by using parallel plate chamber (PTW‐N23342) in a Solid Water phantom at several different depths (0.5, 1.0, 2.0, 3.0, and 4.0 cm). The results are shown in Appendix A A1, A6, for different sized applicators. GafChromic film is useful for accurate measurement of percentage depth dose.^(8)^ Therefore, the EBT3 films were used to verify the parallel‐plate chamber results.

## III. RESULTS

### A. HVL

**Figure 6:**
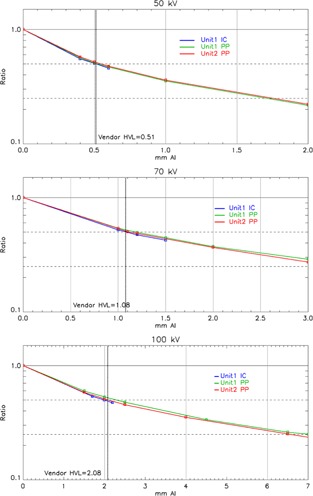
HVL measurements by using different chambers with 2 SRT‐100 units.

The measurements of HVL for two SRT‐100 units with different types of chambers are shown in Fig. 6. The vendor provided HVL values are also included for comparison. Average HVL values for 50, 70, and 100 kV of the two units are 0.52, 1.15, and 2.20 mm Al, respectively. HVL values are 0.06 and 0.33 mm lower if the Farmer chamber is used for 70 and 100 kV, compared with parallel plate chamber measurements (Table 3).

**Table 3:** HVL values (mm AL) measured with two types of chambers for two machines

*Energy (kV)*	*Unit 1 (A11‐TW, vendor)*	*Unit 1, Farmer (PTW‐N23333)*	*Unit 1, PP (PTW‐N23342)*	*Unit 2, PP (PTW‐N23342)*
50	0.51	0.51	0.51	0.53
70	1.08	1.09	1.15	1.15
100	2.08	1.97	2.30	2.10

### B. Dose rate (absolute output)

Dose rates $\left(D_{w.z=0}\right)$ were found to be 3%–4% lower with the Farmer chamber when compared with the PP chamber. This is demonstrated in Table 4. As described in TG‐61, backscatter factors and ratios of energy absorption coefficients (see Table 4) also introduce uncertainty into the dose rate. Although the discrepancy between the values from the two types of chambers are similar to the TG‐61 estimated in‐air measurement standard uncertainty (3.5%), the deviations from backscatter factors and energy absorption coefficient ratios are lower than the TG‐61 estimated uncertainty value of 1.5%. Therefore, the primary sources of discrepancy between the measurements from different chambers are the uncertainty of the calibration factors and the beam flatness. Sources of uncertainty will be addressed further in the Discussions section below. When the same PP chamber was used for the SRT‐100 unit with a similar HVL value, the dose rates were found to be 2%–9% different from the previous unit.

**Table 4:** Dose rate $D_{w,z=0}$ (cGy/min) measured by two chambers at 15 cm TSD under 5 cm applicator in air (ratios of average mass energy‐absorption coefficients water to air and backscatter factors were included)

*Energy (kV)* $D_{w,z=0}$	*Unit 1 (A11‐TW, vendor)*	*Unit 1, Farmer (PTW‐23333)*	*Unit 1, PP (PTW‐23342)*	*Unit 2, PP (PTW‐23342)*
50 kV	748.7	734.4	768.8	783.5
70 kV	662.4	664.8	683.9	728.5
100 kV	644.4	633.9	659.9	711.9
$[({\bar{\mu }}_{en}/{{\rho )}_{air}^{w}]}_{air}$				
50 kV	1.029	1.028	1.028	1.027
70 kV	1.019	1.019	1.019	1.019
100 kV	1.018	1.018	1.019	1.018
B_w_				
50 kV	1.081	1.081	1.081	1.083
70 kV	1.133	1.134	1.137	1.137
100 kV	1.183	1.179	1.190	1.183

### C. Applicator cone factors

As expected, both chambers measured the same applicator cone factors (within 0.3% compared to vendor supplied data), from 0.97 to 1.0 for the 1.5 to 5 cm applicators, as shown in Table 5.

**Table 5:** Applicator cone factors for 1.5 to 5 cm applicators

*Energy (kV)*	*1.5 cm*	*2 cm*	*2.5 cm*	*3 cm*	*4 cm*	*5 cm*
50	0.971	0.978	0.988	0.994	0.998	1.000
70	0.967	0.973	0.987	0.994	0.999	1.000
100	0.961	0.969	0.983	0.993	0.997	1.000

### D. Dose profile for 5 cm applicator

**Figure 7:**
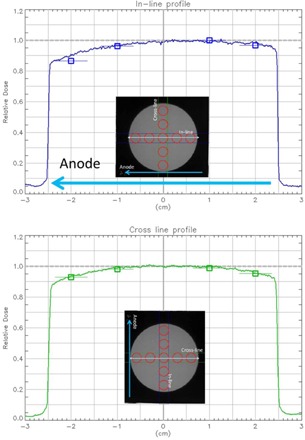
In‐line and cross‐line profile for 50 kV beam with 5 cm applicator.

Figure 7 shows the in‐line and cross‐line profiles. The squares in Fig. 7 are ion chamber measurements at respective locations and the horizontal lines indicate the dimension of the chamber. As shown, the chamber readings agree well with film measurements. Within a 2 cm diameter circle, the dose profile is uniform (<4% deviation); however, the dose decreases as distance from center increases. The nonuniformity is present in both directions. The cross‐line profile is more symmetric than the in‐line dose profile. The lowest dose occurs on the anode side along the X‐ray tube direction. This is expected due to the X‐ray target heel effect.

Film measurements demonstrated a maximum of 15% lower dose, normalized to the center, at the field edge on the anode side, and a maximum of 6% lower dose on the cathode side. The cross‐line profile was relatively more symmetric and there was a maximum of 10% lower dose on both sides of the field. Overall, the uniformity of the beam over a circle of 2 cm diameter was within 4% in all four directions. The ion chamber readings agreed with film measurements within 1%–3%.

### E. Dose outside applicator

The measurements of dose outside the applicator at 1 and 2 cm from the field edge are listed in Table 6. At 1 cm outside the field, the dose dropped to less than 1% for all three energies with the 1.5 cm applicator. The outfield dose increased slightly from 0.4% to 0.9% with increasing energy. At 2 cm from the field edge, the outfield dose dropped 60% to 80% relative to the 1 cm values. This drop also decreased when the energy increased. With a larger applicator, a higher outfield dose was measured. The highest outfield doses at 1 cm were 1.2%, 2.1%, and 2.9% for 50 kV, 70 kV, and 100 k V, respectively. The increase at 1 cm was proportional to the increase in applicator size, regardless of energy.

**Table 6:** Relative dose outside the applicator

*Applicator size*	*50 kV*		*70 kV*		*100 kV*	
	*1 cm*	*2 cm*	*1 cm*	*2 cm*	*1 cm*	*2 cm*
1.5	0.39%	0.08%	0.73%	0.22%	0.94%	0.37%
2.0	0.57%	0.16%	0.95%	0.40%	1.30%	0.55%
2.5	0.70%	0.23%	1.22%	0.49%	1.64%	0.70%
3.0	0.87%	0.31%	1.49%	0.62%	1.98%	0.91%
4.0	1.05%	0.39%	1.88%	0.83%	2.49%	1.17%
5.0	1.18%	0.46%	2.10%	0.96%	2.85%	1.40%

### F. Percentage depth dose

**Figure A3:**
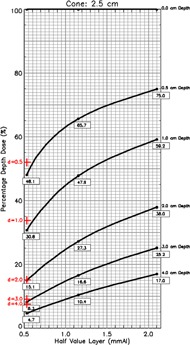
Percentage depth‐dose curves for applicators 2.5 cm dia., 15 cm SSD. EBT3 film measured results for 50 kV at different depth were also included (red cross).

The percentage depth doses for six different sized applicators are listed in Table 7. The PDD curves were also shown in the Appendix A1, A6. For all three energies, PDD increases with increasing applicator size. The PDD increase with applicator size was greater for higher energy. For 50 kV, the increase in PDD was minimal (<1% at all the depths for 1.5 to 5 cm applicators). We used EBT3 film to measure the PDD for 50 kV with the 2.5 cm diameter applicator to verify the PDD measured by the PP chamber. The results are plotted with the ion chamber measurements in Fig. A3 and the film dosimetry agreed well with chamber measurement (within 4% of PDD) at all depths. This agreement was expected as seen in the other study which investigated GafChromic film for low energy X‐ray PDD measurement.^(8)^

**Table 7:** Percentage depth dose

*Depth\diameter* ^a^	*1.5*	*2.0*	*2.5*	*3.0*	*4.0*	*5.0*
*50 kV*						
0.0	100.0%	100.0%	100.0%	100.0%	100.0%	100.0%
0.5	47.2%	47.8%	48.1%	47.8%	47.7%	47.9%
1.0	29.5%	30.6%	30.8%	31.3%	31.3%	31.9%
2.0	14.0%	14.4%	15.0%	15.5%	15.8%	16.2%
3.0	7.4%	7.9%	8.3%	8.6%	9.0%	9.4%
4.0	4.3%	4.5%	4.7%	5.0%	5.3%	5.7%
*70 kV*						
0.0	100.0%	100.0%	100.0%	100.0%	100.0%	100.0%
0.5	65.2%	65.8%	65.7%	65.2%	64.6%	65.1%
1.0	46.5%	48.0%	47.8%	48.6%	48.3%	49.3%
2.0	25.7%	26.4%	27.3%	27.9%	28.5%	29.3%
3.0	15.2%	16.0%	16.6%	17.2%	17.9%	18.8%
4.0	9.5%	10.0%	10.4%	11.0%	11.6%	12.4%
*100 kV*						
0.0	100%	100%	100%	100%	100%	100%
0.5	75%	75%	75%	74%	74%	74%
1.0	58%	60%	59%	60%	60%	61%
2.0	36%	37%	38%	39%	40%	41%
3.0	23%	24%	25%	26%	27%	29%
4.0	16%	16%	17%	18%	19%	20%

^a^ In cm.

## IV. DISCUSSION

The measured HVL values were used to determine the beam quality, back scatter, and applied to derive the air kerma calibration coefficient from the ADCL calibration report. From 1, 2, the parallel plate chamber has a slightly better calibration uncertainty for $N_{k}$ (1%) as opposed to the Farmer chamber (1.5%) in the ADCL reports. In addition, the energy‐dependent $N_{k}$ values of the parallel plate chamber are relatively more uniform than that of the Farmer chamber. A 1 mm difference in HVL of Al results in approximately 2% and 7% of $N_{k}$ uncertainty for PP and Farmer chamber, respectively.

Three different sets of equipment were used to measure the absolute dose for the first unit and all three measurements agreed to within 4%. However, the values measured by the Farmer chamber were 3%–4% lower than those measured by the parallel plate chamber. To investigate this discrepancy, the dose profile of the largest applicator (5 cm) was measured. Figure 7 demonstrates the dose profiles are not flat across the entire field in both the in‐line and cross‐line directions. Although the uniformity over a 2 cm diameter circle is within 4%, any misalignment of the Farmer chamber will underestimate the output due to the size of Farmer chamber (23 mm length). Furthermore, there is no inherent alignment tools or dedicated chamber holder for the Farmer chamber. For such a small TSD setup, it is a challenge to position the Farmer chamber accurately for output measurement as a small deviation can introduce a lager error. In addition, the greater energy dependence of $N_{k}$ makes the Farmer chamber less desirable for the measurement of an X‐ray unit. Therefore, a small‐volume parallel plate chamber is a more appropriate choice as recommended by TG‐61 and other studies with similar dosimetry measurements of low‐energy surface treatment units.^5^, ^9^

Dose outside the applicator falls rapidly as the distance increased. At 1 cm, the outfield dose is less than 1% for 50 kV beam with applicator size smaller than 4 cm. Compared to 6 MeV electron skin treatment, the superficial X‐ray has sharper penumbra and suggests better normal skin sparing.

## V. CONCLUSIONS

The dosimetric data from the commissioning data of two SRT‐100 units were provided in this study. The data obtained from a Farmer chamber underestimates the dose by 3%–4% in comparison to a parallel plate chamber. The discrepancy has been investigated and the results demonstrate the parallel plate chamber is more suitable for this type of measurement. The dose from two identical treatment units with different X‐ray tubes can deviate by up to 9%. The commissioning of a soft X‐ray machine should be done with proper dosimetric methods and equipment.

## Supporting information

Supplementary Material

## Figures and Tables

**Figure A1:**
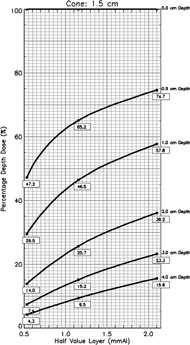
Percentage depth‐dose curves for applicators 1.5 cm dia., 15 cm SSD.

**Figure A2:**
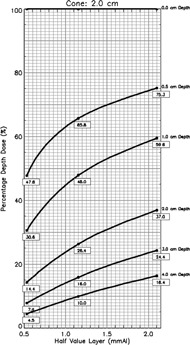
Percentage depth‐dose curves for applicators 2.0 cm dia., 15 cm SSD.

**Figure A4:**
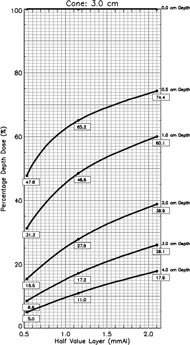
Percentage depth‐dose curves for applicators 3.0 cm dia., 15 cm SSD.

**Figure A5:**
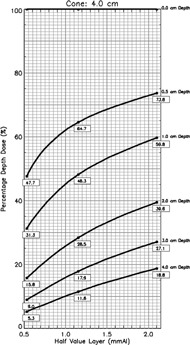
Percentage depth‐dose curves for applicators 4.0 cm dia., 15 cm SSD.

**Figure A6:**
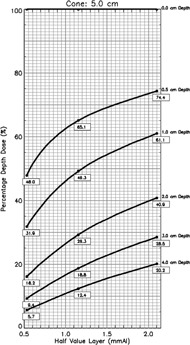
Percentage depth‐dose curves for applicators 5.0 cm dia., 15 cm SSD.
