# Supplementary material for: Commissioning a 50–100 kV X‐ray unit for skin cancer treatment
Source: J Appl Clin Med Phys. 2015 Mar 8;16(2):161–74. doi: 10.1120/jacmp.v16i2.5182 (PMC5690081; doi:10.1120/jacmp.v16i2.5182)
Supplement: Supplementary file 1 — Supplementary Material [file ACM2-16-161-s001.doc]

Associate editor:

This article provides a useful description of the process used to commission two Sensus Healthcare SRT-100 systems. The measurements and results were described in reasonable detail but some key information needs to be added and/or explained more completely.

A: Additional information has been added and the responses are included in detailed comments.

One concern is the calibration of the two ion chambers used to measure the dose rate from the system because the 100 kV chamber sensitivities are extrapolated from calibration values obtained at lower operating voltages.

Also, the calibrations were done with more heavily filtered beams than those from the SRT-100 so the impact on dose rate measurements should be mentioned.

A: The ADCL calibration factors (Nk) for the Farmer chamber are for 0.35,

1.03 and 2.31mm Al HVLs, which encompass the energy range of the Sensus unit. Therefore linear interpolations between these values for the Farmer chamber are acceptable. The calibration factor for the parallel plate chamber has small energy dependence (2% per mm Al). The HVL for the highest operating voltage (100 kV: 2.1mm Al) is only 0.3 mm greater than the calibration value 1.83 mm Al. The uncertainty of the Nk value obtained from the linear extrapolation is within the calibration uncertainty (1%) provided by the ADCL. This has been described in more detail in the discussion section.

Finally, the presentation and comparison of the depth dose curves should be condensed into a larger table and, possibly, two figures to trim down the paper and aid in comparison between the different applicators. The authors need to pay additional attention to grammar in the paper.

A: The PDD table has been added. The PDD figures are moved to the appendix.

Detailed comments

Line 24 This final statement in the abstract is not mentioned in the

body of the paper or the conclusion.

A: The final statement in the abstract has been revised to indicate the disadvantage of using a Farmer-type chamber to measure the SRT-100 x-ray treatment unit.

Line 67 … as well as six five applicators …

A: The number of applicators has been corrected to six.

Line 90 Data collected during commissioning …

A: It has been corrected.

Line 91 This article presents dose rate at 15 cm for the 5 cm

applicator; were dose rates collected for the other applicators as well?

A: The dose rate, i.e., absolute output was measured for only the 5 cm applicator. The dose rates for other applicators were calculated using applicator cone factors. The “absolute output” was added to the Materials and Methods section.

Line 93 It appears that only HVL and dose rate at 15 cm for the 5 cm

cone are compared with vendor data in this paper. If you compared all of your measurements with vendor data, it would be valuable to provide more information.

A: Since the dose rates for other cones were calculated by cone factors and they were within 0.3% compared to Vendor’s numbers for different units, the only difference is absolute output of 5 cm applicator. Therefore, we didn’t list the full comparison table for all the applicators.

Line 98 Please note how you extrapolated the chamber calibrations to

100 kV. Since each chamber only had two calibration points was it simply a linear extrapolation?

A: Linear extrapolation was used for the PP chamber and a linear

interpolation was performed to obtain the Farmer Nk values. Please see

Comment 2.

Line 100 Please explain what you mean by ‘user determined values’?

A: We meant the HVL’s we derived from measurement. The paragraph has been revised.

Line 119 Please replace ’… kV energy’ with ‘… kV operating

voltage’.

A: The kV energy now is revised as kV operating voltage.

Line 129 Please add information on your measurement technique used to

determine relative dose outside of the applicator.

A: A description has been added after line 129.

Line 149 Please provide a bit more information on the 2-9% dose rate

difference between units. Was this due to measurement uncertainty or that the x-ray output differed for the same beam current?

A: Since reproducibility for 3 separate measurements was within 3%, there

must be an another reason for the 2-9% variation in output. We do not have

the resources to analyze the electrical components of the Senus unit, but we suspect the difference in dose rate results from variation in the x-ray tube output due to a variation in beam current.

Line 174 This section will be more meaningful with information added on

the measurement locations (see comment above for Line 129)

A: The description of measurement setup and condition has been added.

Line 183 This section would be more effective if the figures could be

condensed. It is difficult for the reader to compare between cone sizes so a table with numerical values would help support the conclusions. (An example of a table is added at the end of these comments.) It would also be helpful to note the measurement reproducibility compared to the variation seen between cones.

A: Table 7 was added. The graphical display of PDD vs HVL might be still useful as a quick reference. Figures 8 to 13 were changed to appendix (A1 to A6).

Line 189 The agreement appears to be within 4 percentage points of APDD

but the numerical values seem to differ by 10-50% between film and the PP chamber.

A: It has been revised and specified as 4% of PDD to avoid confusion.

Line 198 Please explain the comment about ‘a 1 mm difference in HVL’

more clearly.

A: Since Nk is a function of the HVL, a larger HVL dependence magnifies the uncertainty of the Nk measurement. If we plot the normalized Nk vs. HVL, it is apparent that the parallel plate chamber (N23342) is less energy dependent (2% per mm Al HVL) than the Farmer chamber (7% per mm Al HVL).

Line 215 It would be helpful to comment on your comparisons with

vendor-supplied data in the Discussion section.

A: We requested out of field dose information from the vendor but it was not supplied.

Journal of Applied Clinical Medical Physics http://www.jacmp.org
